# Unusual presentation of childhood Systemic Lupus Erythematosus

**DOI:** 10.1186/1546-0096-5-20

**Published:** 2007-11-21

**Authors:** Sathish Kumar, Indira Agarwal

**Affiliations:** 1 Department of Child Health Unit II, Christian Medical College, Ida Scudder Road, Vellore 632 004, India

## Abstract

Bullous systemic lupus erythematosus is a rare blistering condition with a distinctive combination of clinical, histological and immunopathologic features that together constitute a unique bullous disease phenotype. It is often associated with autoimmunity to type VII collagen. Here we report a child who presented with bullous systemic lupus erythematosus. Rapid resolution of the blisters occurred following treatment with dapsone.

## Introduction

Bullous systemic lupus erythematosus (SLE) is a rare, distinctive subepidermal blistering disorder that occurs in systemic lupus erythematosus [1]. It is characterized clinically by a pemphigoid-like eruption with tense fluid-filled vesicles and bullae, often with a background of maculopapular or urticated erythema. It can affect any area of the body, including non-sun-exposed sites and the mucous membranes. Pruritus is usually present in variable severity. The lesions form erosions and crusts before healing, usually but not invariably without scarring. Treatment with dapsone results in promising results. We describe a 13 years old girl who presented with bullous SLE.

## Case presentation

A 13-year-old girl presented with recurrent fever associated with increasing fatigue, arthraliga, hair loss and erythematosus bullous lesions over face, neck and extremities of 2 months duration. She also had one episode of generalised tonic clonic seizures prior to admission.

**Figure 1:**
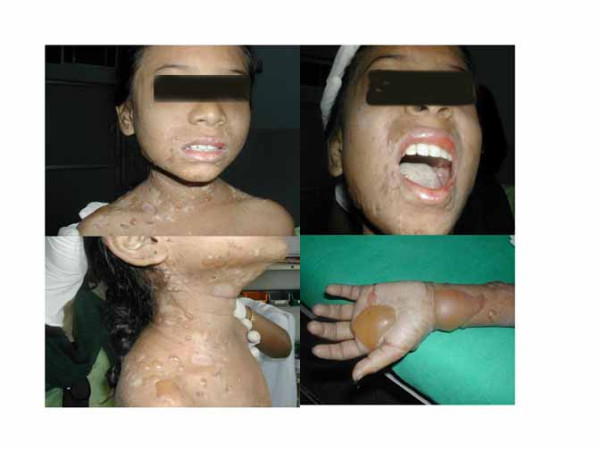
SLE child with urticarial, erythematous eruption associated with tense blisters, erosions, and crusting over face, upper chest and upper extremities.

She was treated with oral antibiotics and topical hydrocortisone for her skin lesions. On examination she had numerous bullae and vesicles on a background of urticated plaques affecting the back, abdomen, neck and flexures of the arm and groin regions (Fig. 1). Nikolsky's sign was negative. Erosions with crusts characterized older lesions. No scarring was seen at the sites of healed lesions. There were no mucosal erosions or blisters. No malar rash or oral ulcers. No raynaud's phenomenon. Cardiovascular and respiratory system examination were within normal limits. Abdominal examination did not reveal hepatosplenomegaly. Her fundoscopy did not reveal evidence of hypertensive encephalopathy or vasculitis. Otherwise neurologically she was normal.

Investigations revealed hemoglobin was 90 g/L, white blood cell was 21 × 10^9^/L with neutrophils of 2.5 × 10^9^/L, lymphocytes of 1 × 10^9^/L, bands 0.03 × 10^9^/L. Her platelets were 20 × 10^9^/L. Her INR and PTT were within normal limits. ESR was 55 mm at 1 hour and CRP was negative. Her serum creatinine was 42 μmol/L. ANA was positive in 1/640 titers with speckled pattern and DsDNA was elevated. Complements (C3 and C4) were low. Anticardiolipin antibody and lupus anticoagulant were negative. Anti-Ro, anti-La, anti-Sm antibodies were also negative. Urine microscopy showed microscopic hematuria and proteinuria. 24 hour urinary protein was 20 mg/m^2^/hr. CSF analysis was within normal limits including opening pressure.

Skin biopsy from these bullous lesions revealed a neutrophil-predominant inflammatory infiltrate in the upper dermis with dermal-epidermal separation. Direct immunofluorescence (DIF) showed prominent fluorescence along the epidermal basement membrane for IgG, IgA, IgM and C3. There was also smudgy fluorescence rimming some of the dermal blood vessels for IgA, IgM & C3. She was diagnosed to have Bullous SLE with renal and CNS involvement. Renal biopsy was deferred in view of thrombocytopenia.

She was treated with prednisolone 2 mg/kg/day and dapsone 50 mg for her skin lesions. She was also administered cyclophosphamide 500 mg/m2 as infusion with hydration. Her skin lesions improved within a week. As parents wanted to try alternative medicine, she was discharged at request.

## Discussion

Bullous systemic lupus erythematosus (BSLE) is a rare, chronic, non-scarring blistering eruption, characterized by subepidermal blisters with acute predominantly neutrophilic inflammation in the upper dermis, immune complex linear deposition at the basement membrane by immunofluorescence, and immune deposits beneath the lamina densa by ultrastructural analysis [2]. Less than 5% patients with SLE develop vesiculobullous lesions in isolation or in addition to other cutaneous manifestations [3].

Blisters in SLE can be due to bullous SLE or SLE with blisters. Histologically, bullous SLE is a subepidermal blistering disease with an acute neutrophil-predominant infiltrate in the upper dermis. In contrast, the histology of cutaneous lesions of SLE with blistering reveals severe edema in the upper dermis and hydropic degeneration of the basal layer. Epidermal necrosis is seen at the advancing edges of the lesions [4].

DIF of bullous SLE demonstrates linear or granular deposition of IgG (with or without IgA and/or IgM) and complement deposition at the basement membrane zone. Indirect immunofluorescence (IIF) of serum may demonstrate circulating antibodies to type VII collagen. These antibodies usually demonstrate binding to the dermal side of salt-split skin preparations on IIF. Our child did not have IIF. These antibodies are thought to be pathogenic because type VII collagen is a major component of the anchoring fibril, which has a central role in the generation of basement membrane-dermal adhesion. It is thought that immune complexes binding to type VII collagen or complement mediated damage to type VII collagen impairs anchoring fibril function and leads to subepidermal blister formation [5].

The precise delineation of bullous SLE from several primary blistering disorders (eg, bullous pemphigoid, dermatitis herpetiformis, pemphigus vulgaris, epidermolysis bullosa acquisita) is based on [1] widespread cutaneous vesiculobullous eruption [2] histology of skin lesions demonstrating acute neutrophilic upper dermal infiltrate and subepidermal separation [3] a positive direct or indirect immunofluorescence test demonstrating antibodies directed against the basement membrane [4] a tendency to respond to treatment with dapsone and [5] the presence of autoantibodies to type VII collagen as seen in epidermolysis bullosa acquisita (EBA).

Dapsone is the mainstay of treatment of bullous SLE [6]. Bullous lupus often responds dramatically to dapsone, usually with ceasing of the formation of new lesions within 12–48 h after initiation of therapy and with healing of old lesions within several days. Relatively low doses (25–50 mg daily) may be efficacious in some cases. Rapid recurrences may occur upon withdrawal of dapsone, with prompt remission after reinstitution of therapy [7]. Methotrexate is also efficacious in management of bullous SLE [8]. Azathioprine, antimalarials and cyclophosphamide have been used as steroid-sparing agents in those cases unresponsive to dapsone. The disease usually remits, often within 1 year.
